# Expression profile analysis of long non-coding RNA in skeletal muscle of osteoporosis by microarray and bioinformatics

**DOI:** 10.1186/s13036-019-0180-5

**Published:** 2019-05-31

**Authors:** Shaojin Liu, Hongxing Huang, Shuang Chai, Hewei Wei, Jiachun Huang, Lei Wan

**Affiliations:** 1 Guangzhou University of Chinese Medicine, Guangzhou, 510405 China; 2 Department of Orthopaedics, The Third Affiliated Hospital, Guangzhou University of Chinese Medicine, Guangzhou, 510378 China; 3 Lingnan Medical Research Center of Guangzhou University of Chinese Medicine, Guangzhou University of Chinese Medicine, Guangzhou, 510405 China

**Keywords:** lncRNA, Microarray profile, Musculoskeletal, Osteoporosis, Quantitative real-time PCR

## Abstract

**Background:**

Osteoporosis (OP) is a condition featured by bone mass loss and bone tissue microarchitectural alterations due to impaired tissue homeostasis favoring excessive bone resorption versus deposition. The trigger of such an impairment and the downstream molecular pathways involved are yet to be clarified. Long non-coding RNA (lncRNA) plays a role in gene transcription, protein expression and epigenetic regulation; and altered expression results in immune or metabolism related desease development. To determine whether lncRNAs are involved in osteoporosis, we analyzed the expression profile of lncRNAs and mRNAs in osteoporosis.

**Method:**

Three pairs of osteoporosis patients (OP group) and healthy people controls (NC group) were screened by microarray. Quantitative polymerase chain reaction (qRT-PCR) was performed to confirm dysregulated lncRNA expressions in 5 pairs of OP and NC group tissues samples. Gene Ontology (GO) and Kyoto Encyclopedia of Genes and Genomes (KEGG) pathway analyses were performed to construct the lncRNA-mRNA co-expression network.

**Result:**

Through co-expression analysis, differently expressed transcripts were divided into modules, and lncRNAs were functionally annotated. We further analyzed the clinical significance of crucial lncRNAs from modules in public data. Finally, the expression of five lncRNAs, CUST_44695_PI430048170-GeneSymbol:CTA-384D8.35;CUST_39447_PI430048170,CUST_73298_PI430048170,CUST_108340_PI430048170,CUST_118927_PI430048170,this four lncRNAs have not been annotation genes and have not found GeneSymbols, and by quantitative RT-PCR, which may be associated with osteoporosis patients’ overall survival.

**Conclusion:**

Analysis of this study revealed that dysregulated lncRNAs and mRNAs in osteoporosis patients and health people controls could affect the immune or metabolism system and musculoskeletal cell differentiation. The biological functions of those lncRNAs need to be further validated.

## Background

Osteoporosis (OP), is a systemic skeletal disorder, characterized by decreased bone mass, deterioration of the microarchitecture of bone tissue and increased fragility as well as consequent increase in risk of bone fracture, which greatly affects people’s life quality and even gives rise to the increased mortality, arousing extensive concerns among the population [1]. Osteopenia is less severe and refers to bone density that is below normal peak density but not low enough to be classified as osteoporosis [2].

Our understanding of bone-muscle crosstalk has been historically based on mechanical interactions between the bone and muscle. The bone is shaped by mechanical force applied by muscles, and the bone provides an attachment site for the muscle to maintain shape and drive locomotion [3]. The mechanical aspects of bone-muscle interactions are critical for normal development and movement and play a large role in changes of these tissues in disease and aging, yet the interactions between the bone and muscle are more complicated [4]. Just as our understanding of other organ system integrations has advanced, so too has our understanding of the complex endocrine-based crosstalk between the bone and muscle [3]. Bone and muscle anabolism are tightly coupled during growth and development. Conversely, bone and muscle catabolism occur during aging. Compromising either the bone or muscle by disease, disuse or aging affects both tissues but the cellular and molecular mechanisms linking these are not well understood [5]. Skeletal muscle and bone mass are influenced by various factors, including diet and nutrition, genetics, hormones, growth factors and mechanical stimuli [6]. The mass of bone and skeletal muscle is maintained in proportion to the mechanical loading experienced by the musculoskeletal system. For example, resistance training leads to an increase in muscle size and bone mineral density, which is associated with improved musculoskeletal fitness [7].

Long non-coding RNAs (lncRNAs) with length longer than 200 nucleotides are defined as transcripts that are not translated into protein [8]. Long non-coding RNA (lncRNA) regulates gene transcription and protein expressions genetically and epigenetically, and altered expressions result in immune or metabolism related desease development [9]. Among these newly discovered RNA elements, lncRNAs have been identified to have functional roles in a diverse range of cellular functions such as development, differentiation, cell fate, as well as disease pathogenesis [10]. Among the ncRNAs, two of them received lately extensive attention: miRNAs and lncRNAs. Several review the role of miRNAs and/or lncRNAs in adult skeletal myogenesis and muscle diseases [5]. LncRNAs can be divided into five broad categories: sense, antisense, bidirectional, intronic, and intergenic [11]. They are involved in diverse biological process and pathogenesis of disorders, such as osteoblast differentiation, osteosarcoma,and cardiovascular [12]. However, to our knowledge, little is known about lncRNAs expression profile in related to skeletal and muscle, and the potential pathways regulating osteoporosis remain poorly understood [13].

This pilot study aimed to identify aberrantly expressed lncRNAs and mRNAs profile of muscle disease and explore their potential functions in osteoporosis. In particular, using public databases, we identified the clinical significance of lncRNAs, it may be useful as diagnostic and prognostic biomarkers and provide novel therapeutic targets in related to skeletal and muscle diseases of the osteoporosis.

## Materials and methods

### Patients and tissue samples

A total of 3 pairs of osteoporosis and health patients skeletal muscle tissues were surgically obtained from adult patients undergoing treatment at The Third Affiliated Hospital, Guangzhou University of Chinese Medicine (Guangzhou,China) between January 2017 and March 2018. The detailed characteristics of the study subjects are summarizedin Table 1. All the tissues were collected in anterior cruciate ligament reconstruction with autogenous tendon and fracture surgery patient. The fresh muscle were achieved from operating room and processed immediately in liquid nitrogen within 15 min and then storage in RNA Fixer Reagent (Bioteke,Beijing,China) at− 80^∘^C prior to total RNA extraction. 3 pairs of tissues underwent microarray analysis and the remaining 10 tissues were used in validation studies by quantitative real-time polymerase chain reaction (qRT-PCR).

**Table 1:** Characteristics of the study subjects

Number of tissues	Gender	Age (years)	Height (cm)	Weight (kg)	Lumbar T-score
NC-1	Male	33	176	78	−1.2
NC-2	Male	30	167	63	1.4
NC-3	Male	60	170	66	1.4
OP-1	Male	43	178	80	−3.4
OP-2	Female	52	156	58	−3.7
OP-3	Female	68	152	46	−2.8

The Ethics Committee in Clinical Research of The Third Affiliated Hospital, Guangzhou University of Chinese Medical approved this study, and written informed consent was provided by all patients.

### Transcript analysis

#### RNA extraction and purification

Total RNA was extracted using TAKARA RNAiso Plus#9109 following the manufacturer’s instructions and checked for a RIN number to inspect RNA integrity by an Agilent Bioanalyzer 2100 (Agilent technologies, Santa Clara, CA, US). Qualified total RNA was further purified by RNeasy micro kit (Cat#74004, QIAGEN, GmBH, Germany) and RNase-Free DNase Set (Cat#79254, QIAGEN, GmBH, Germany).

#### RNA amplification and labeling

Total RNA was amplified and labeled by Low Input Quick Amp Labeling Kit, One-Color (Cat.#5190–2305, Agilent technologies, Santa Clara, CA, US), following the manufacturer’s instructions. Labeled cRNA were purified by RNeasy mini kit (Cat.# 74,106, QIAGEN, GmBH, Germany).

Each slide was hybridized with 1.65 μg Cy3-labeled cRNA using Gene Expression Hybridization Kit (Cat.# 5188–5242, Agilent technologies, Santa Clara, CA, US) in Hybridization Oven (Cat.# G2545A, Agilent technologies, Santa Clara, CA, US), according to the manufacturer’s instructions. After 17 h hybridization, slides were washed in staining dishes (Cat.# 121, Thermo Shandon, Waltham, MA, US) with Gene Expression Wash Buffer Kit (Cat.# 5188–5327, Agilent technologies, Santa Clara, CA, US), followed the manufacturer’s instructions。.

### Data acquisition

Slides were scanned by Agilent Microarray Scanner (Cat#G2565CA, Agilent technologies, Santa Clara, CA, US) with default settings, Dye channel: Green, Scan resolution = 3 μm, PMT 100%, 20bit. Data were extracted with Feature Extraction software 10.7 (Agilent technologies, Santa Clara, CA, US). Raw data were normalized by Quantile algorithm, limma packages in R. We slect a standard threshold set for differentially expressed genes of a fold change≥2.0 and a 푝 value≤0.05.

### Gene ontology and Kyoto encyclopedia of genes and genomes pathway analyses

Gene Ontology (GO) analysis was applied to analyze the main function of the differential expression genes according to the GO database. Pathway analysis was used to find out the significant pathway of the differential genes according to KEGG.

### lncRNA-mRNA coexpression networks

R function cor.test (a test for association/correlation between paired sam-ples) was utilized to compute Pearson’s correlation coeficient to measure the gene coexpression. The lncRNAs (fold change≥2.0, value≤0.01 and length 0,10,000),mRNAs (fold change≥4.0 and 푝 value≤0.01) were choosed to draw the network by Cytoscape.

According to these data, we built lncRNA-mRNA network using the correlation coefficients to examine interactions between lncRNA and mRNA. The value of “degree” in coexpression network indicated that one mRNA/lncRNA might be correlated with several lncRNAs/mRNAs.

### qRT-PCR analysis

Total RNA was extracted and purified using standard methods (Life Technologies; RNA Easy, Qiagen, Valencia, CA,USA). Bestar qPCR RT Kit reverse transcription (Promega) was utilized to synthesize cDNA. 5 lncRNA (fold change≥2.0, value≤0.01 and length 0,3000 ChrX) expressions in sinonasal tissues were measured by qRT-PCR which was performed on the ABI7500 qPCR system with the primer pairs listed in Table 2. The raw quantifications were normalized to the beta-actin gene values for each sample and fold changes were shown as mean ± SD in three independent experiments, each in triplicate.

**Table 2:** Real-time quantitative PCR primer sequences used in this study

Probe Name	Forward primers (5′-3′)	Reverse primers (5′-3′)	Amplicon size (bp)
CUST_39447_PI430048170	GTTCCCGAGGTGTCCGA	TCGTTAGTCGAGCTGAAACC	148
CUST_44695_PI430048170	CGGTGAGGTAGAAGGAGAAC	TCTTCCTCAGCAGCACTTCC	159
CUST_73298_PI430048170	AATCCTTGGCATTGTCTGTAA	AGCCAGTAGACGACACCAGC	149
CUST_108340_PI430048170	GAACAAAGGAGCAACAGCCC	CCTGATAGGAATCTGCGAAAA	83
CUST_118927_PI430048170	GAGGCTGGAATGCGGAGT	CACCTACTTTCCCTTGGCTATT	160

### Statistical analysis

All data were expressed as the mean ± SD or proportions where appropriate. Expression levels skeletal muscle tissues from osteoporosis patients were analyzed by paired-sample푡-tests.푝values < 0.05(two-tailed) indicated statistical significance. The Statistical Program for Social Sciences (SPSS) 20.0 software was employed to perform all of the statistical analyses.

## Results

### Differentially expressed lncRNAs and mRNAs in osteoporosis

**Fig. 1:**
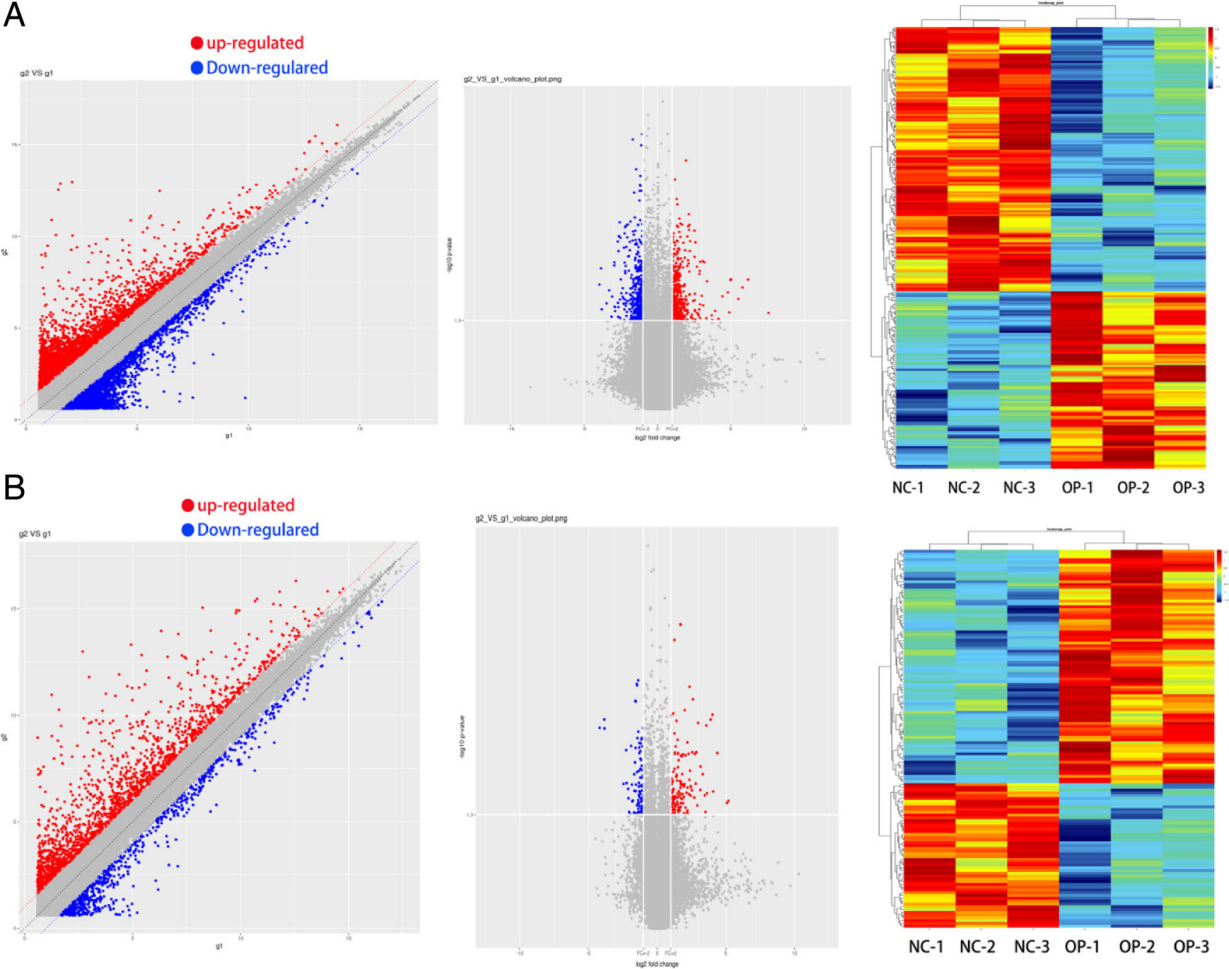
Scatter Plot,Volcano plots and heat map showing expression profiles of long non-coding RNAs (lncRNAs) (**a**) and mRNAs (**b**) in osteoporosis patinas (OP group) and normal healthy people (NC group). Three plots are based on the expression values of all lncRNAs and mRNAs detected by microarray. These maps showing significantly changed lncRNAs and mRNAs with fold change ≥2.0 respectively (P < 0.05; false discovery rate < 0.05)

Volcano plots were used for assessing gene expression variation between osteoporosis and health patient groups. In total, 1594 lncRNAs displayed differential expression in osteoporosis, including 482 upregulated lncRNAs and 1112 down regulated lncRNAs. Of 886 mRNAs that showed differential expression, 436 were upregulated and 450 were downregulated. Among them, 94 lncRNAs and 84 mRNAs were significantly upregulated, and 140 lncRNAs and 52 mRNAs were significantly downregulated> 2-fold in osteoporosis. Hierarchical clustering analysis showed systematic variations in the expression of lncRNAs and mRNAs among samples. The data suggested that the expression of lncRNAs and mRNAs in osteoporosis differ from those in health people controls (Fig. 1). As expected, the lncRNA and mRNA expression proiles were distinguishing osteoporosis and normal tissue samples accurately based on the molecular signature.

Hierarchical clustering of the lncRNAs and mRNAs proile was performed using cluster 3.0.2; hierarchical clustering of the expression of the dysregulated lncRNAs and mRNAs based on centered Pearson correlation clearly separated osteoporosis tissues from corresponding normal tissues (Fig. 1). Out of the group of RNAs that were upregulated, lncRNA CUST_35058_PI430048170 and mRNA CUST_56753_PI430048170 showed the greatest degree of demonstrated upregulation, with 72.21419842-and 36.48758725-fold increases, respectively; of those that were downregulated, lncRNA CUST_21632_PI430048170 and mRNA CUST_139172_PI430048170 demonstrated the greatest degree of downregulation, with 15.62387857-and 18.19379213-fold decreases, respectively (Tables 3 and 4).

**Table 3:** Top 20 aberrantly expressed lncRNAs in microarray for 3 pairs of osteoporosis patients and health people skeletal muscle tissues

ProbeName	P	FC (abs)	Regulation	GeneSymbol	Accession	Chr
CUST_35058_PI430048170	0.012556283	72.21419842	up	MIR503HG	ENST00000440570	chrX
CUST_35059_PI430048170	0.016349269	55.40531579	up	MIR503HG	NR_024607	chrX
CUST_126144_PI430048170	0.024118939	45.69384441	up	MIR503HG	NR_024607	chrX
CUST_126145_PI430048170	0.012357324	32.40658055	up	MIR503HG	ENST00000457876	chrX
CUST_35062_PI430048170	0.012723529	31.4726076	up	MIR503HG	ENST00000457876	chrX
CUST_109345_PI430048170	0.038064776	29.70049534	up	SAA3P	NR_026576	chr11
CUST_41271_PI430048170	0.022023032	21.58734413	up	RP11-283C24.1	ENST00000578585	chr17
CUST_21632_PI430048170	0.01486414	15.62387857	down	VLDLR-AS1	NR_015375	chr9
CUST_110886_PI430048170	0.036123995	15.25447339	up	RUNX1-IT1	NR_026812	chr21
CUST_98863_PI430048170	0.00857309	14.40142299	down	–	lnc-RASA1–18:1	chr5
CUST_80467_PI430048170	0.034308762	13.89512258	down	VLDLR-AS1	NR_015375	chr9
CUST_56635_PI430048170	0.049061646	13.26382976	up	–	lnc-PTEN-11:1	chr10
CUST_112522_PI430048170	0.042135672	12.93582703	up	RP11-283C24.1	ENST00000578585	chr17
CUST_10165_PI430048170	0.028081239	11.13188848	down	VLDLR-AS1	NR_015375	chr9
CUST_71013_PI430048170	0.013220249	10.40135012	up	–	lnc-INSM1–5:3	chr20
CUST_113140_PI430048170	0.046258578	10.05254523	up	–	lnc-CCDC144NL-4:1	chr17
CUST_124876_PI430048170	0.0180434	9.786534613	down	AC003090.1	ENST00000446840	chr7
CUST_21634_PI430048170	0.044021797	8.77824946	down	VLDLR-AS1	NR_015375	chr9
CUST_71788_PI430048170	0.030034472	8.664571816	up	–	lnc-RCAN1–6:1	chr21
CUST_19829_PI430048170	0.022251937	8.525312141	down	RP11-797H7.5	ENST00000340779	chr7

**Table 4:** Top 20 aberrantly expressed mRNAs in microarray for 3 pairs of osteoporosis patients and health people skeletal muscle tissues

ProbeName	P	FC (abs)	Regulation	GeneSymbol	Accession	Chr
CUST_56753_PI430048170	0.0348289	36.48758725	up	SCD	NM_005063	chr10
CUST_133279_PI430048170	0.036740847	33.94274666	up	SAA1	NM_000331	chr11
CUST_141790_PI430048170	0.009996764	20.86176002	up	SAA4	NM_006512	chr11
CUST_139172_PI430048170	0.005145352	18.19379213	down	SBK2	NM_001101401	chr19
CUST_141387_PI430048170	0.027240985	16.90436771	up	SCD	NM_005063	chr10
CUST_135342_PI430048170	0.003639094	16.28422029	up	MRAP	NM_178817	chr21
CUST_130964_PI430048170	0.004130947	14.9015582	up	SAA4	NM_006512	chr11
CUST_138578_PI430048170	0.004133096	14.36030139	down	AFP	NM_001134	chr4
CUST_145430_PI430048170	0.005228882	14.17959829	down	PLCH1	NM_001130961	chr3
CUST_132601_PI430048170	0.028566771	12.39092273	up	DGAT2	NM_032564	chr11
CUST_128647_PI430048170	0.004831104	11.96333249	up	PXDNL	NM_144651	chr8
CUST_136078_PI430048170	0.046626016	11.41446117	up	ANKRD1	NM_014391	chr10
CUST_139601_PI430048170	0.030211027	11.00284852	up	ADIPOQ	NM_001177800	chr3
CUST_142975_PI430048170	0.017278415	10.02050967	up	PNPLA3	NM_025225	chr22
CUST_126930_PI430048170	0.014106518	8.930113104	up	LGALS12	NM_001142537	chr11
CUST_142712_PI430048170	0.026107426	8.824194597	up	RYR3	NM_001036	chr15
CUST_130312_PI430048170	0.004510607	7.885502819	up	KLB	NM_175737	chr4
CUST_52015_PI430048170	0.039281946	7.576008123	up	RYR3	NM_001036	chr15
CUST_141047_PI430048170	0.009777851	7.231495622	up	RBP4	NM_006744	chr10
CUST_136229_PI430048170	0.010210594	6.756306455	up	TUSC5	NM_172367	chr17

### Functional analysis of differentially expressed genes

**Fig. 2:**
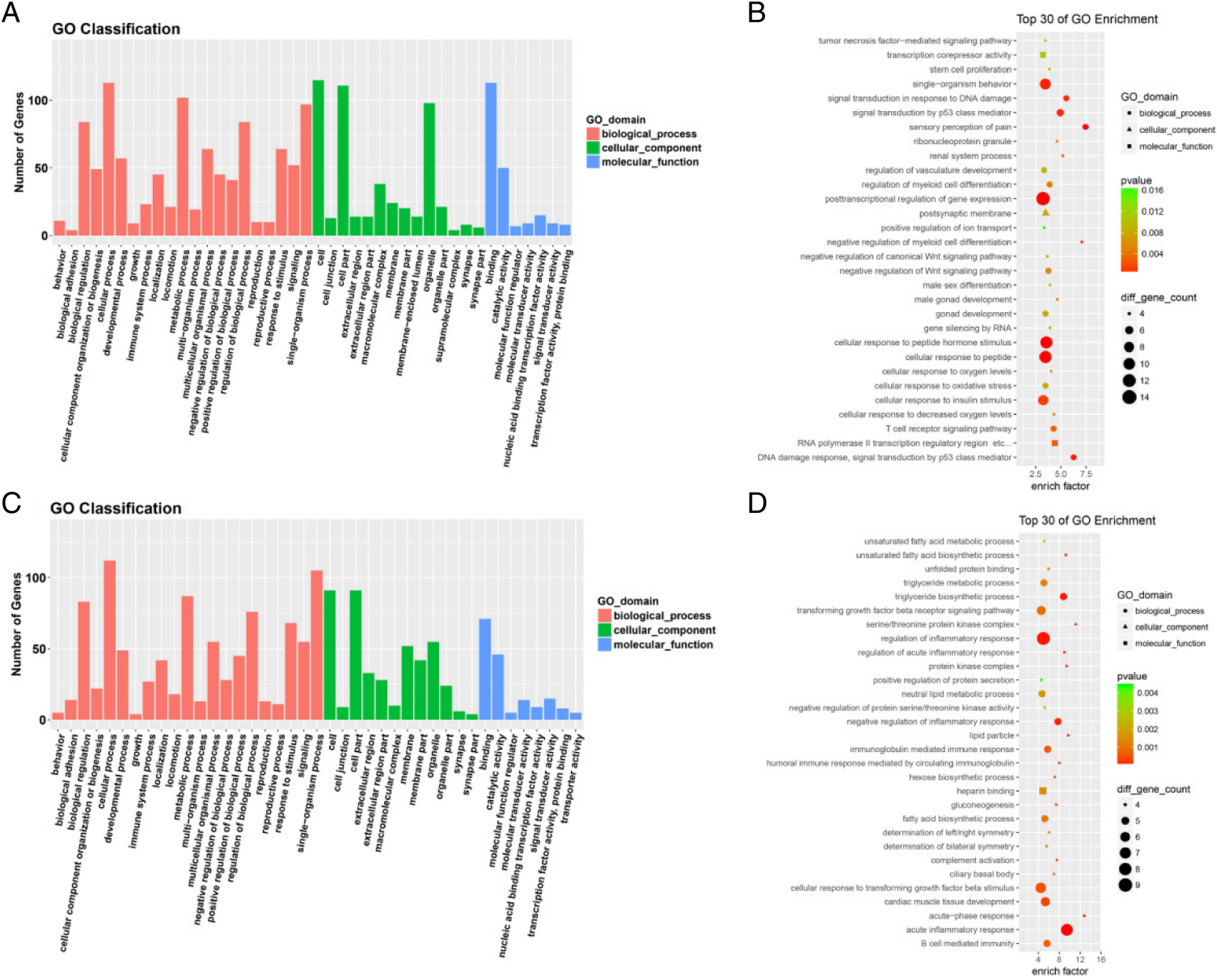
GO analysis. Functions of significantly differently expressed lncRNAs,mRNAs (fold change> 2; p < 0.05) were analyzed by GO annotations, and functions of novel lncRNAs were deduced by their co-expressed mRNAs. The GO database includes 3 parts (cellular component, biological process and molecular function) that together describe the genes and gene products across all species. **a** and **c**, GO terms of biological process, cellular component,and molecular function of lncRNAs,mRNAs. **b** and **d**, GO annotations of up-and down regulated lncRNAs and mRNAs with top 30 enrichment scores of biological processes

**Fig. 3:**
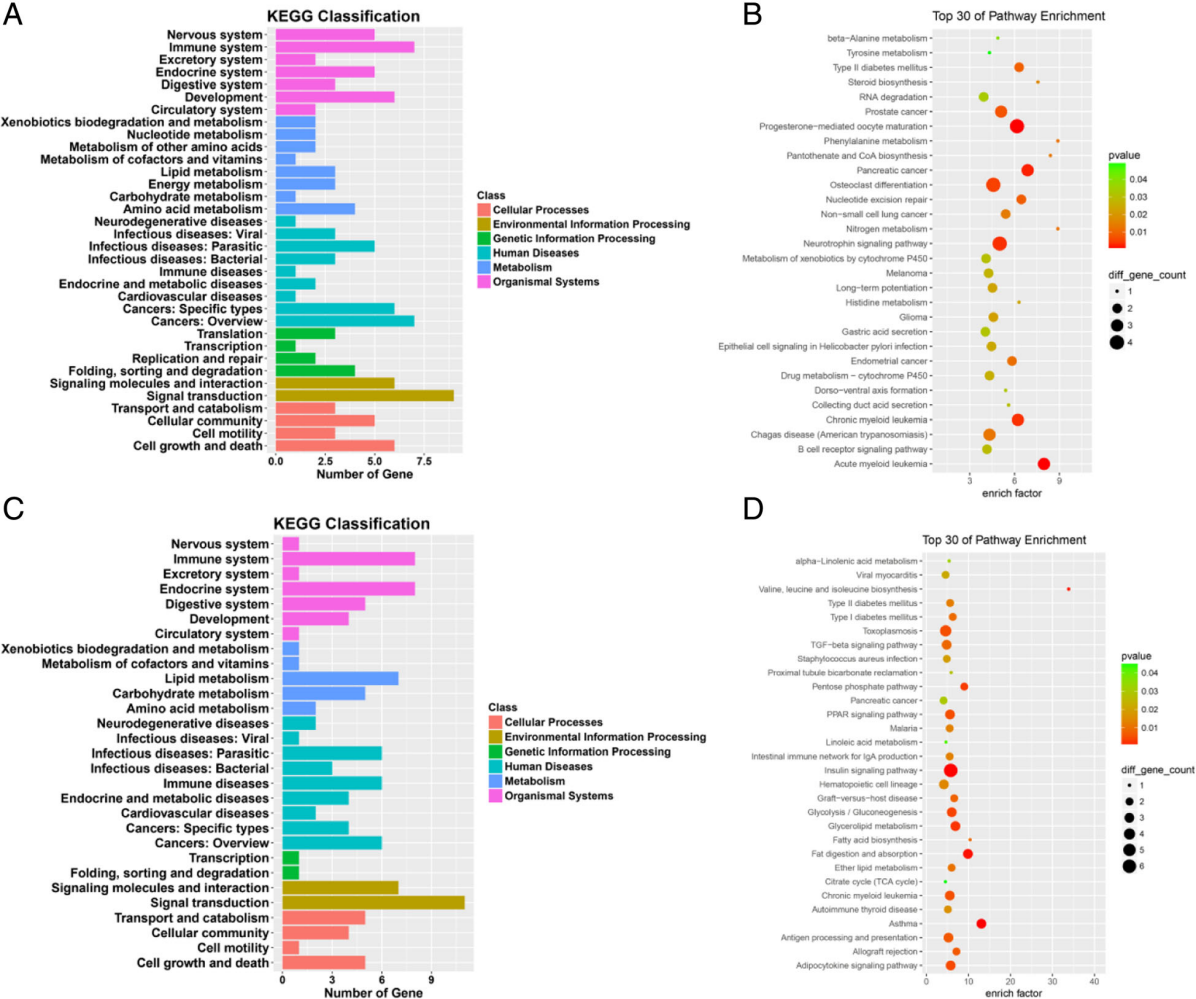
Kyoto Encyclopedia of Genes and Genomes (KEGG) pathway analyses. The KEGG pathway is a collection of manually drawn signal pathway maps and provides a valuable tool for mapping a specific gene to its corresponding pathway. **a** and **c**, KEGG classification of Cellular Processes, Environmental Information Processing, Genetic Information Processing, Human Diseases, Metabolism and Organismal Systems of lncRNAs,mRNAs. **b** and **d**, KEGG pathway enrichment analysis scores of up-and down regulated lncRNAs and mRNAs with top 30

Until now, the functions of most lncRNAs have not been well annotated. Therefore, by analyzing differentially expressed mRNAs, we can forecast the role that lncRNAs play in osteoporosis. The GO and KEGG pathway analyses of differentially expressed lncRNAs and mRNAs could provide a clue about the osteoporosis disease process. We utilized all differentially expressed mRNAs for the GO analysis and found that the most enriched GO targeted by upregulated and downregulated transcripts were involved in anterior/posterior pattern specification, immune or metabolism system processes, and immune response (Fig. 2). In the KEGG pathway analysis, the down-and up-regulated mRNAs were found to be mostly enriched in muscle tissues, respectively (Fig. 3). Many genes involved in muscle tissues differentiation were dysregulated. A pathway network was constructed using 20 of the most significantly enriched pathways to illustrate the critical pathways in the process of osteoporosis. The bone metabolism pathway were considered to be the most central functions in the net because the exchanges with other pathways strongly depended on their existence (Fig. 3).

### Long non-coding RNA/mRNA coexpression network in osteoporosis

**Fig. 4:**
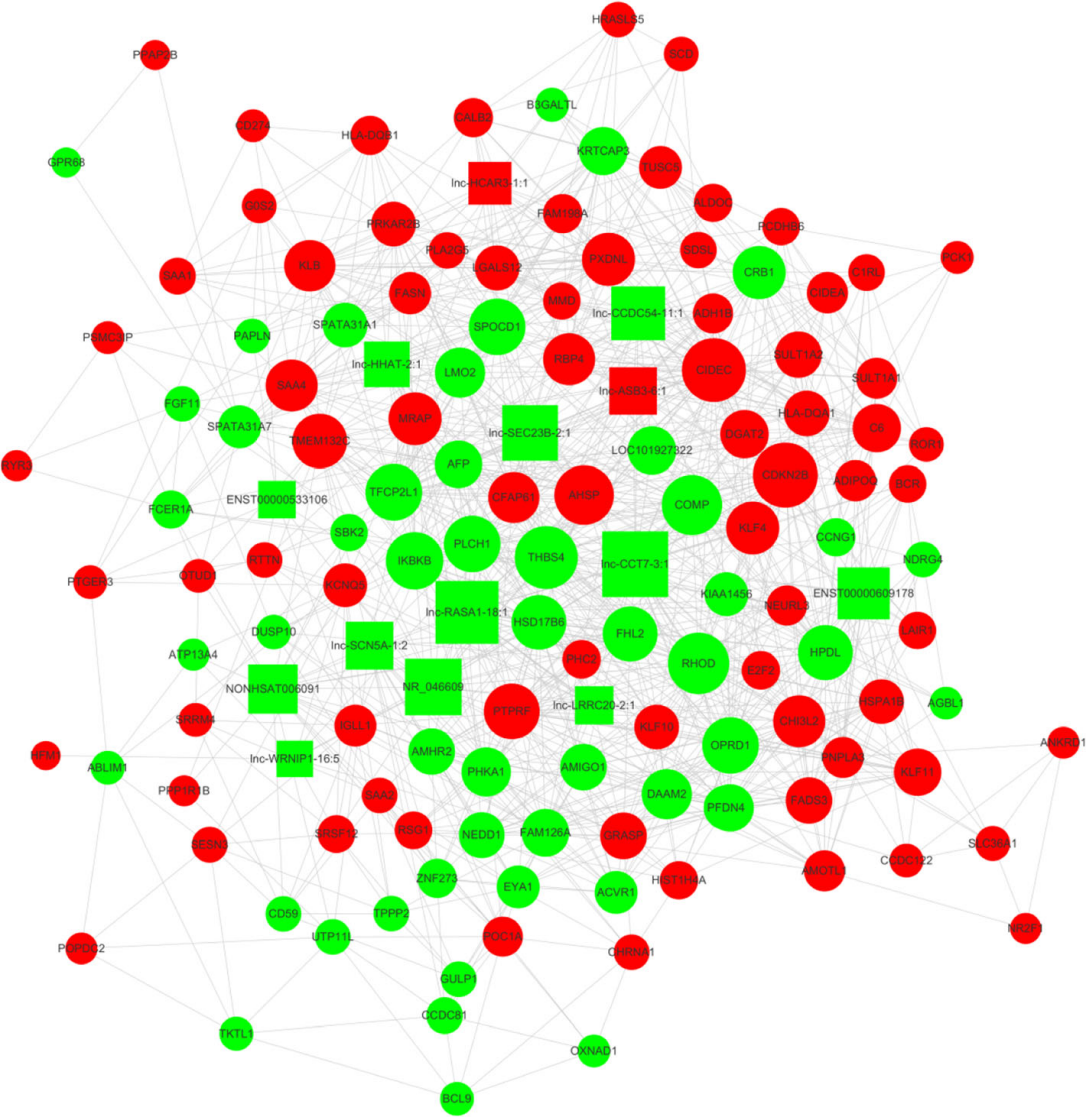
The lncRNA-mRNA co-expression network. The osteoporosis patients (OP) consisted of coexpression relationships between lncRNAs and mRNAs. The circles denote mRNAs and the square denote lncRNAs((green: downregulated genes; red: upregulated genes). The node degree is indicated by the circle size. An edge represents a coexpression relationship between mRNA and a lncRNA in the context of OP progression. Data were analyzed and constructed by Cytoscape software (The Cytoscape Consortium). This co-expression network suggests an inter-regulation of lncRNAs and mRNAs in OP development

We constructed the lncRNA-mRNA coexpression network to identify the interactions between mRNAs and lncRNAs. Transcriptome regulation involves a huge network, among which many transcripts form a complex web to function. Coexpression networks facilitate the intricate network based on gene screening methods that can be used to identify candidate biomarkers or therapeutic targets. The most crucial subnetwork was constructed by the transcripts with a high *k*-score, which would be the core regulatory modules of the entire coexpression network (Fig. 4). What is more, the transcripts in this network are widely distributed in all chromosomes, indicating the widely interconnected regulation network between lncRNAs and mRNAs (Fig. 4). This subnetwork includes four lncRNAs,lnc-CCT7–3:1,lnc-RASA1–18:1,NR_046609,and lnc-SEC23B-2:1, constituting probably the core of the network. The results implied that mRNA CDKN2B,CIDEC,and THBS4 may play key roles in osteoporosis process and development.

### qRT-PCR validation

**Fig. 5:**
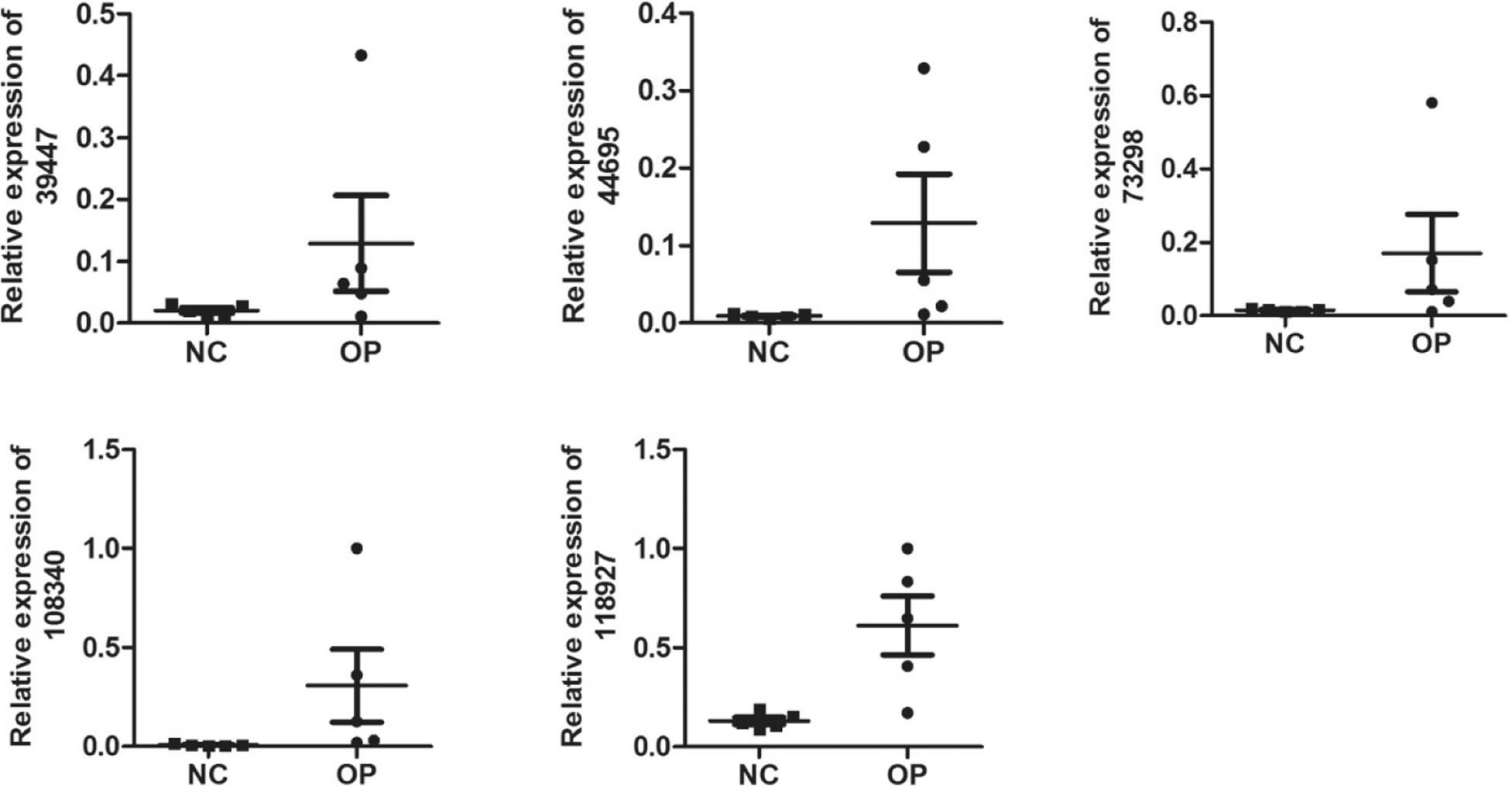
qRT-PCR validation. qRT-PCR verification of 5 candidate lncRNAs in 5 pairs of OP and NC group tissue. Expression of pediatric OP samples vs. control samples was analyzed using qRT-PCR, and summarized as mean average ± standard error (SE). *P* < 0.05 was considered statistically significant

5 differentially expressed lncRNAs were randomly selected for validation by means of qRT-PCR according to the manufacturer’s recommendations. CUST_108340_PI430048170 was upregulated and CUST_39447_PI430048170,CUST_44695_PI430048170,CUST_73298_PI430048170,and CUST_118927_PI430048170 were downregulated in osteoporosis. The results of qRT-PCR were consistent with those of the microarray. All of the 5 lncRNAs were differentially expressed with the same trend (up-or downregulated) (Fig. 5).

## Discussion

It is well known that the mutation of genes and chromosomes contribute to the pathogenesis of leukemia. However, lncRNAs, the rising stars in biology, have just begun to be understood, and the majority of them have not yet been researched [14]. To provide some insights into the biological functions of lncRNAs in the pathogenesis of osteoporosis, we undertook a comprehensive analysis of lncRNA and mRNA profiling data from osteoporosis patients and health people, together with data from a public database. We identified the core lncRNAs and their functional annotations, and validated their expression by qRT-PCR. Overall, our work uncovered an interlaced transcripts network that is involved in osteoporosis development, in which lncRNAs play an indispensable role.

We explored the expression patterns of transcripts between osteoporosis patients and health people controls’ muscleskeletal. Microarray data identified vast lncRNAs and mRNAs, supporting an extensive involvement of lncRNAs in osteoporosis. There were two important concepts that ran throughout our studies to handle the mass data. First, we simplified the complex transcript network by modularization. The GO and KEGG pathway analyses divided mRNAs into several functional modules, which are related to immunity, metabolism, osteoclast differentiation, hematopoiesis, and TGF-beta signaling pathway, indicating the validity of the microarray [15]. Cytoscape was used to construct co-expressed networks from the public and our microarray data, respectively, and then the networks were facilitated into several sub-networks through the k-score method. In the same way, lncRNAs were attributed their correlated functional mRNA modules through the co-expressed network and GO annotations. Overall, modularization contributes to simplifying the intricate network into modules, which were like “big genes”. Further analysis of public data showed that the lncRNA may regulate osteoclast differentiation, leading to the decrease bone mass, cause to osteoporosis happened [16]. Finally, five lncRNAs, CUST_39447_PI430048170,CUST_44695_PI430048170,CUST_73298_PI430048170,CUST_108340_PI430048170,and CUST_118927_PI430048170 were confirmed as significantly differentially expressed in osteoporosis patients and healthy controls by qRT-PCR.

Our work clearly indicated an important role for lncRNAs in OP. However, many lncRNAs were excluded as they failed to be allocated to functional modules and have not been included in public data. It was difficult to originally understand the functions and targets of these lncRNAs, which may also play a key role in osteoporosis [17]. In addition, our analysis showed that lncRNA lnc-CCT7–3:1 was highly correlated with osteoporosis, its neighboring gene. The lncRNA may be directly or indirectly correlated with osteoporosis and there may be additional transcripts involved in the lncRNA-associated biological process [18]. The lncRNA’s biological functions need to be validated further.

The last decades witnessed the discovery of biological functions for non-coding RNA, which triggered the recognition that RNA is not only a simple hinge of the central dogma but also directly takes part in the regulation of biological networks [19]. With the development of next-generation sequencing, especially in terms of depth and scale, significant data has been accumulated. We have to recognize the system is so complex that it is beyond the initial recognition. Fortunately, the progress of methodology simplifies the networks. Through modularization, thousands of transcripts can be facilitated into several “big genes,” and then the core lncRNAs of each module can be researched in details. Moreover, the active application of accumulated public data will help us to make the functions of lncRNAs more clear. Hopefully, this study can provide a reference for the broad analysis of OP data.

Skeletal muscle possesses a remarkable ability to adapt to various physiologic conditions [20]. AMPK is a sensor of intracellular energy status that maintains energy stores by fine-tuning anabolic and catabolic pathways [21]. At the same time, skeletal muscle has been shown to be important for regulating whole-body metabolism. Skeletal muscle demonstrates high malleability and can adapt its contractile composition and metabolic properties in response to a number of physiologic conditions [22]. However, bone mass and skeletal muscle mass are controlled by factors such as genetics, diet and nutrition, growth factors and mechanical stimuli. Whereas increased mechanical loading of the musculoskeletal system stimulates an increase in the mass and strength of skeletal muscle and bone, reduced mechanical loading and disuse rapidly promote a decreasein musculoskeletal mass, strength and ultimately performance [26]. Moreover, skeletal muscle atrophy often occursinparallel tobonelossand activity; musclemassand strength influence bone mass [23]. However, because the mechanisms that regulate mechanotransduction in bone and muscle are complex, it is not entirely clear which specific mechanisms operate in synergy during musculoskeletal disuse [24]. Treatment options for muscle disuse atrophy and osteoporosis are currently limited, with rehabilitative strength training proving to be the most effective method to restore musculoskeletal mass; however, long periods of rehabilitation are necessary to restore muscle, bone and locomotor performance following artificial immobilisation in non-hibernating mammals [25]. Muscules and bones are the main part of the motor system, regulating processes such as endocrine and metabolism [26]. IGF-1 in muscle tissue promotes bone development by improving muscle mass and strength, and also directly promotes bone development, increasing bone mineral content and bone density [27]. Myostatin is most abundantly expressed in skeletal muscle tissue, inhibits muscle growth and regeneration, and promotes osteoclast differentiation, inhibiting TNF-α-induced osteoclast differentiation [28]. Indian Hedgehog (Ihh), a signaling molecule secreted by chondrocytes and osteoblasts, promotes muscle growth. Ihh in bone can directly promote muscle growth [29]. Osteocalcin (Glu-OC) partially repairs muscles with impaired function. The gap junction protein Connexin43 in bone cells directly regulates muscle growth and function [30]. The close connection between bone and muscle is not only related to the mechanics of its interaction, but also to the complex and precise endocrine regulation and some common molecular signaling pathways [31]. The new recent study that significant differences in the expression of lncRNAs, mRNAs, circRNAs, and miRNAs between postmenopausal OP patients and healthy controls, and the functions of the RNAs were also identified based on our RNA-seq analysis [32]. Taken together, our study indicated that lncRNAs and mRNA could associate with the occurrence of OP and may be as possible biomarkers and target genes in OP.

## Conclusions

Insummary, to our knowledge, our study analysis of lncRNA expression profile in osteoporosis. The results show that genes regulated by these lncRNAs are involved in TGF-beta signaling pathways as a proof of principle, or influence osteoclast differentiation. This may offer new insights into pathogenesis and could be a promising way to dissect the molecular pathogenesis of this refractory bone metabolism desease. Our study lays the foundation for further investigation of this disease. Further large scale studies are warranted to provide convincing evidence for clarifying the functions of lncRNAs in osteoporosis and determining whether these lncRNAs can serve as new diagnostic biomarkers, prognostic factors for survival, and therapeutic targets in osteoporosis.

## Abbreviations

GO: Gene Ontology
KEGG: Kyoto Encyclopedia of Genes and Genomes
LncRNA: Long non-coding RNA
OP: Osteoporosis
qRT-PCR: Quantitative polymerase chain reaction
